# Importance of Consistent Datasets in Musculoskeletal Modelling: A Study of the Hand and Wrist

**DOI:** 10.1007/s10439-017-1936-z

**Published:** 2017-10-02

**Authors:** Benjamin Goislard De Monsabert, Dafydd Edwards, Darshan Shah, Angela Kedgley

**Affiliations:** 1 Department of Bioengineering, Imperial College London, Royal School of Mines Building, London, SW7 2AZ UK; 2 Royal Centre for Defence Medicine, Birmingham, UK

**Keywords:** Clinical imaging, Motion capture, Digitization, Instantaneous helical axes, Sarcomere length, Moment arms, Tendon excursions, Muscle force

## Abstract

Hand musculoskeletal models provide a valuable insight into the loads withstood by the upper limb; however, their development remains challenging because there are few datasets describing both the musculoskeletal geometry and muscle morphology from the elbow to the finger tips. Clinical imaging, optical motion capture and microscopy were used to create a dataset from a single specimen. Subsequently, a musculoskeletal model of the wrist was developed based on these data to estimate muscle tensions and to demonstrate the potential of the provided parameters. Tendon excursions and moment arms predicted by this model were in agreement with previously reported experimental data. When simulating a flexion–extension motion, muscle forces reached 90 N among extensors and a co-contraction of flexors, amounting to 62.6 N, was estimated by the model. Two alternative musculoskeletal models were also created based on anatomical data available in the literature to illustrate the effect of combining incomplete datasets. Compared to the initial model, the intensities and load sharing of the muscles estimated by the two alternative models differed by up to 180% for a single muscle. This confirms the importance of using a single source of anatomical data when developing such models.

**Electronic Supplementary Material:**

The online version of this article (doi:10.1007/s10439-017-1936-z contains supplementary material, which is available to authorized users.

## Introduction

The hand is essential for daily living. It is required for many vital tasks, e.g., eating, and is used during both forceful and fine manipulation tasks, e.g., hammering and sewing. Musculoskeletal disorders affecting the hands, such as osteoarthritis, can thus be dramatically debilitating. However, our limited understanding of hand biomechanics hinders the development of appropriate prevention, treatment, and rehabilitation methods. The hand is comprised of 27 bones and requires more than 50 muscles for the actuation of all the joints. It is not only technically difficult to track the motion of all these segments, but also impossible to directly measure the forces exerted through all the muscles and joints of the hand. Nevertheless, these data are needed to understand normal hand function and the effect of disorders on this.

Sensors have been developed to obtain direct *in vivo* measurements of forces exerted in finger flexor tendons^12^ but, for ethical and technical reasons, they cannot be used to measure the simultaneous actions of the numerous hand muscles. Physiological joint simulators have been able to determine the muscle load sharing required to replicate wrist motions *in vitro*,^23^,^27^ but the complexity of designing such systems, and the limitations of cadaveric testing, prevent the generalization of such approaches. Musculoskeletal models use computational representations of the anatomy to investigate the biomechanical behaviour of complex structures, such as the hand. These models can be used to solve the mechanical equations of motion and estimate the muscle forces required to accomplish a task.^12^

Many musculoskeletal models have been developed to study a single finger^5^,^9^,^22^,^25^ or several fingers independently.^21^ Although these models provide valuable insights into hand biomechanics, they neglect the wrist, which is critical for hand function. The extrinsic hand muscles originate in the forearm; their tendons cross the wrist before inserting onto the phalanges. These muscles therefore act simultaneously about the joints of the fingers and the wrist, creating a direct mechanical coupling between these joints. Recent models that included this coupling resulted in more physiologically realistic simulations, including estimating co-contraction,^13^,^20^ which corroborates results from electromyographic studies. To expand these models, anatomical datasets describing the entire musculoskeletal system from the humerus to the tips of the fingers are needed, but these are rare. Models that include both the fingers and the wrist^13^,^20^ use a combination of different anatomical datasets corresponding to different specimen populations, potentially creating inconsistencies.

Chao *et al*.^7^ have provided an extensive anatomical dataset, comprising the three-dimensional (3D) coordinates of points describing the trajectories of all the muscles and tendons about the finger joints. At the wrist, others have established the relationships between muscle/tendon moment arms and joint angles for the wrist prime movers^14^,^18^ and other muscles.^4^ However, these relationships were functionally measured using joint displacement and tendon excursion^2^ rather than using the positions of points along the muscle/tendon path.^7^ Hence, although data are available for the fingers and wrist, the types of data and calculation methods differ. Finally, for none of the datasets mentioned above is the morphology of the muscles of the specimens provided. These parameters have been measured, but only for certain muscle groups, such as the wrist prime movers^16^ or the hand extrinsic muscles.^17^

The main objectives of this study were (1) to obtain a complete anatomical dataset, providing a description of the musculoskeletal system of the forearm and the hand, including muscle/tendon geometrical paths and muscle morphological parameters, (2) to design a wrist musculoskeletal model using the collected dataset and estimate muscle forces during a simple wrist motion to demonstrate the potential of the model and (3) to demonstrate the effect of combining different anatomical datasets in the same musculoskeletal model. We hypothesized that the different datasets would result in different estimations of the muscle forces and load sharing.

## Methods

### Collection of the Anatomical Dataset

The dataset was collected from the left forearm of a fresh-frozen cadaveric specimen (male, age: 53 years, weight: 115 kg, height: 183 cm) resected at the mid-diaphyseal level of the humerus. Ethical approval for the use of this specimen was obtained from the Tissue Management Committee of the Imperial College Healthcare Tissue Bank, according to the Human Tissue Act. Prior to the dissection, gross anthropometric measurements of the specimen were taken.

#### Musculoskeletal Geometry

**Figure 1:**
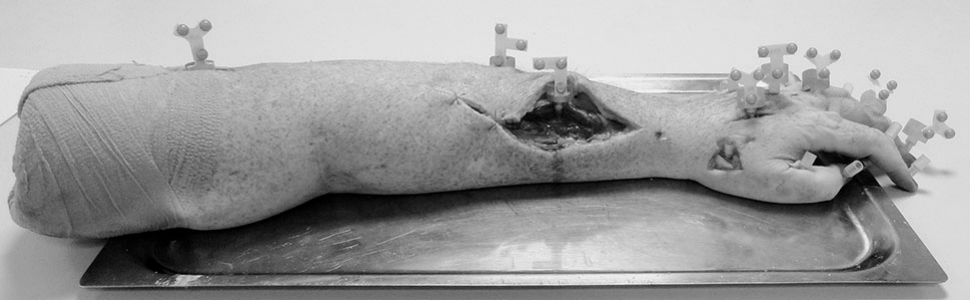
Forearm specimen equipped with all the 22 cluster bases. The eight clusters are mounted on the humerus, the ulna, the radius, the second and third metacarpals, and the phalanges of the middle finger.

A 6-camera optical motion capture system (Oqus 5 +, residual error < 0.3 mm, Qualisys, Sweden) was used to track the positions of eight clusters, each comprised of three markers (Fig. 1), and a digitisation wand, comprised of five markers. Owing to the technical challenges of simultaneously tracking all 22 segments, the clusters were designed to be mounted and dismounted on different segments. An M4 ball and spring plunger system ensured accurate and repeatable cluster placement. A digitisation wand was used to digitise anatomical points, lines and surfaces on the specimen (error < 0.45 mm, as determined during the wand calibration).

Initial dissection included the excision of skin, fat and connective tissues to reveal the bone surface to which the cluster bases were affixed. The bases were secured to each segment with self-tapping wood screws and care was taken to not impinge any tendons or ligaments. Six static trials were initially recorded, one for the forearm and one for each finger, to define a reference pose where the position of all clusters was known. The third metacarpal (MC3) cluster was used as the reference coordinate system. The error associated with the transformations of the marker clusters to the reference posture^24^ was less than 0.2 mm. All geometrical measurements presented correspond to this reference pose and are expressed in the anatomical coordinate system of the left ulna.^29^

Bony landmarks were digitized directly on the skin of the forearm and hand to define coordinate systems for all segments.^29^ The joints were passively moved through uniaxial motions to estimate the location and orientation of functional axes using the instantaneous helical axis method.^26^ The specimen was then dissected progressively to enable the digitisation of the origin, insertion and *via* points, representing the path of each muscle/tendon. *Via* points were digitised to describe more precisely the paths of the tendons between their origin and insertion points. These points were especially chosen to represent the paths of tendons when crossing a joint, e.g., entrance and exit to retinaculum, and to characterize the interconnection between finger tendons, e.g., extensors. After the dissection, the wand was used to digitise dorsal surfaces of the finger joints about which the tendons wrapped. Simple geometrical shapes, i.e., cylinders or spheres, were fit to these traces to further constrain the tendon paths in the resultant musculoskeletal model.

#### Muscle Morphological Parameters

After dissection, each muscle/tendon unit was transfixed to a wooden board and fixated in a 4% formalin solution. The muscle belly length, fibre length, tendon length and pennation angle were measured using a metal ruler and protractor. Depending on the size of the muscle, 2–5 readings of fibre length and pennation angle were taken at different locations; these were averaged to obtain a single value for each muscle. The tendons were carefully resected off the musculotendinous junctions to measure muscle mass using a digital scale (accuracy: 0.1 g).

To obtain the optimal muscle fibre length of each muscle, the sarcomere length of the muscle in its fixated state was measured using digital microscopy. For each muscle, two fibre samples (10–50 fibres, 2 cm long), one close to the origin of the fibres and one close to the insertion, were dissected and conserved in a 50% glycerol solution. From each sample, smaller samples consisting of 1–5 fibres were obtained under a dissection microscope (EZ4D, Leica Microsystems, UK) at 35 times magnification; these were mounted onto glass slides using glycerol solution and a glass cover slide. Digital images (257 *μ*m at 500 times magnification) of these fibre samples were taken using a camera (AxioCam ICc 1, Zeiss, Germany) mounted on a light microscope (Axio Scope.A1, Zeiss, Germany). Each image was processed in MATLAB (The Mathworks, Natick, MA) using a custom-written programme to calculate the sarcomere length. Images were converted to greyscale format to obtain the intensity profile along a manually drawn line, parallel to the fibre. Maximal intensity peaks were detected and used to count the number of sarcomeres in the profile (mean 38.7 ± 11.1) and calculate the sarcomere length. The final sarcomere length of a muscle was obtained by averaging the estimations obtained for all the images taken for that muscle (between 5 and 17). The ratio of the final sarcomere length to the optimal sarcomere length of 2.7 *μ*m^19^ was used to scale the muscle fibre length to obtain the optimal fibre length.

The physiological cross-sectional area (PCSA) of each muscle was calculated using the equation

1 $${\text{PCSA}}_{m} = \frac{{m_{m} }}{{\rho_{m} \cdot l_{\text{f}}^{0} }},$$

where *m*_*m*_ is the muscle mass, *ρ*_*m*_ the muscle density, assumed to be 1.056 g cm^−3^,^16^ and *l*_f_^0^ the optimal fibre length.

#### Bone Surfaces

Prior to its dissection, the specimen was scanned using computed tomography (CT; SOMATOM Definition AS, SIEMENS, Germany; in-plane resolution = 0.63 mm, slice thickness = 1 mm). 3D surface models of the bones were obtained after segmentation and smoothing operations (MIMICS, Materialise, Leuven, Belgium).

### Wrist Musculoskeletal Model

**Figure 2:**
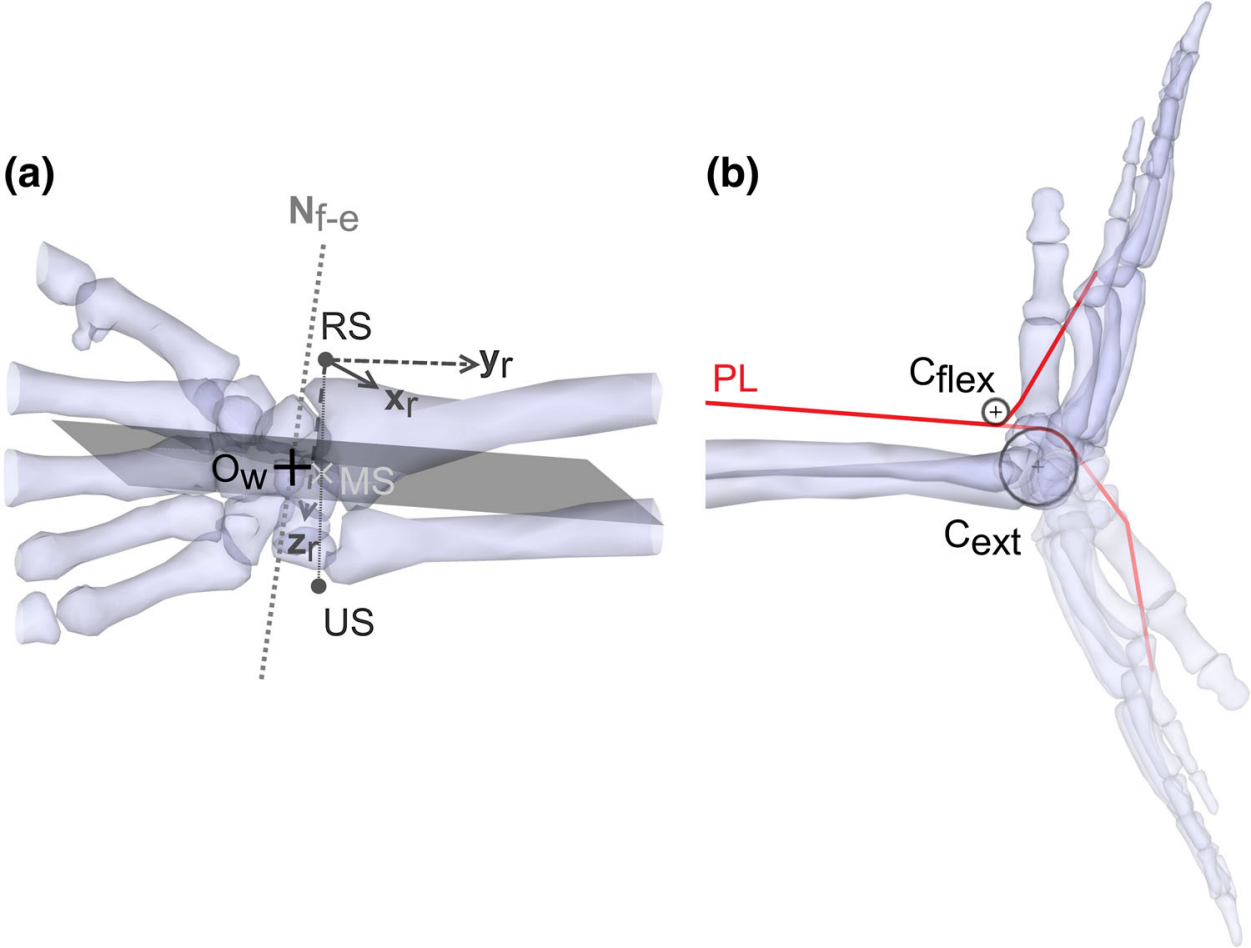
(a) Dorsal view of the wrist model showing the position of the wrist joint centre (*O*_*w*_) with the geometrical elements that were used to determined its position. *N*_*f*-*e*_ is the line representing the flexion–extension axis of the wrist; RS and US are the radial and ulnar styloids. MS is the midpoint between the two styloids; *x*_*r*_, *y*_*r*_, *z*_*r*_ are the axes of the radius coordinate system, calculated as recommended by the ISB.^29^ The shaded plane is orthogonal to the *z*_*r*_ axis and passes through MS. *O*_*w*_ is at the intersection between this plane and *N*_*f*-*e*_. (b) Ulnar view of the wrist model showing the cylinders used to constrain the muscle/tendon path of the palmaris longus (PL) muscle when the joint is in flexion (*C*_flex_) and when it is in extension (*C*_ext_).

A musculoskeletal model of the wrist was created based on the anatomical dataset. The wrist was modeled as two hinge joints between the radius and MC3, one representing radial–ulnar deviation and one representing flexion–extension. The axes were oriented and positioned identically to the corresponding functional axes (Table 4), as determined using the passive joint motion data (see “Musculoskeletal geometry” section). The joint centre of rotation was assumed to be the point at the intersection of the flexion–extension axis and the plane parallel to the sagittal plane of the radius, defined using ISB definitions,^29^ and containing the midpoint between the radial and ulnar styloids (Fig. 2a). Only the prime movers of the wrist, i.e., those muscles inserting on the metacarpals, were considered: flexor carpi ulnaris (FCU), flexor carpi radialis (FCR), palmaris longus (PL), extensor carpi ulnaris (ECU), extensor carpi radialis longus (ECRL), extensor carpi radialis brevis (ECRB) and abductor pollicis longus (APL).

#### Tendon Path

In addition to the origin, insertion and *via* points for each muscle, wrapping objects were created to constrain muscle/tendon paths. Each path was constrained by two cylinders (Fig. 2b), one representing the carpal bones and the other representing the retinaculum.^3^ All cylinders were fixed relative to the radius, with their axis parallel to the wrist flexion–extension axis. Their position along the radius longitudinal and antero-posterior axes, and the their radii, were manually adjusted to obtain both physiologically realistic trajectories, and tendon excursion and moment arm curves consistent with the literature.^4^,^14^,^18^

The paths and lengths of the tendon elements were then calculated using the shortest path method.^8^ The line of action for each muscle was represented by a unit vector directed from the insertion point to either a *via* point proximal to the wrist or to the most distal point wrapping on a cylinder. Moment arms for each tendon about the wrist joint were calculated as the cross-product between the unit vector and the vector going from the wrist joint centre to the insertion point.

#### Estimation of Muscle Forces

The forces exerted by the seven muscles were estimated by solving the moment mechanical equilibrium about the two degrees of freedom of the wrist. Since the system is indeterminate, i.e., more unknowns than equations, there are an infinite number of muscle force combinations to balance the moments at the wrist. An optimisation procedure based on the minimisation of muscle stress^10^ was therefore used to select a solution. The direction of the joint reaction force was also constrained in order to avoid unrealistic estimations,^6^ such as those resulting in a dislocation of the wrist. This optimisation was implemented as follows:

For each time sample, find *f*_*m*_ that minimizes

2 $$g\left( {f_{m} } \right) = \sum\limits_{m} {\left( {\frac{{f_{m} }}{{PCSA_{m} }}} \right)^{2} }$$

and subject to

3 $$\left[ {{\mathbf{I}}_{\text{h}} } \right]{{\vec{\ddot{\varvec{\theta}}}}} = {\vec{\mathbf{M}}}_{\text{p}} + {\vec{\mathbf{M}}}_{\text{e}} + \sum\limits_{m} {{\vec{\mathbf{r}}}_{m} .f_{m} }$$

4 $$0 \le f_{m} \le {\text{PCSA}}_{m} \cdot \sigma_{\hbox{max} }$$

5 $$\alpha_{\hbox{min} } \le \arctan \left( {\frac{{{\vec{\mathbf{R}}}_{{\mathbf{j}}} \cdot {\vec{\mathbf{x}}}_{\text{radius}} }}{{{\vec{\mathbf{R}}}_{{\mathbf{j}}} \cdot {\vec{\mathbf{y}}}_{\text{radius}} }}} \right) \le \alpha_{\hbox{max} }$$

6 $$\beta_{\hbox{min} } \le \arctan \left( {\frac{{{\vec{\mathbf{R}}}_{{\mathbf{j}}} \cdot {\vec{\mathbf{z}}}_{\text{radius}} }}{{{\vec{\mathbf{R}}}_{{\mathbf{j}}} \cdot {\vec{\mathbf{y}}}_{\text{radius}} }}} \right) \le \beta_{\hbox{max} } ,$$

where *f*_*m*_ is the muscle force of muscle *m*, PCSA_*m*_ the physiological cross-sectional area of muscle *m*, [**I**_*h*_] the inertia matrix of the hand at the wrist joint centre, ${{\vec{\ddot{\varvec{\theta}}}}}(t)$ the angular acceleration of the hand centre of mass, ${\vec{\mathbf{M}}}_{\text{p}}$ the moment due to passive constraints in the wrist joint, ${\vec{\mathbf{M}}}_{\text{e}}$ the moment due to the external forces applied to the hand, ${\vec{\mathbf{r}}}_{m}$ the moment arm vector of muscle *m*, *σ*_max_ the maximum muscle stress, ${\vec{\mathbf{R}}}_{j}$ the joint reaction force at the wrist. ${\vec{\mathbf{x}}}_{\text{radius}}$, ${\vec{\mathbf{y}}}_{\text{radius}}$, and ${\vec{\mathbf{z}}}_{\text{radius}}$ are the local coordinate system axes the radius calculated as recommended by the ISB.^29^ *α*_min_, *α*_max_, *β*_min_, and *β*_max_ are the angles used to constrain the components of the joint reaction force corresponding to shear forces; these were equal to 30° (dorsal), 60° (palmar), 85° (radial), and 30° (ulnar direction), respectively. These values were determined using the position of joint surfaces in the bone surface models. The joint reaction force was calculated using the equation

7 $$m_{\text{h}} \cdot {\vec{\mathbf{a}}}_{\text{h}} = {\vec{\mathbf{F}}}_{\text{e}} + \sum\limits_{m} {{\vec{\mathbf{u}}}_{m} .f_{m} } + {\vec{\mathbf{R}}}_{j} ,$$

where *m*_h_ is the mass of the hand, ${\vec{\mathbf{a}}}_{\text{h}}$ the acceleration of the hand centre of mass, ${\vec{\mathbf{F}}}_{\text{e}}$ the resultant force due to the external forces applied to the hand, *f*_*m*_ and ${\vec{\mathbf{u}}}_{m}$ the muscle force and the unit vector describing the line of action of muscle *m*, ${\vec{\mathbf{R}}}_{j}$ the joint reaction force vector.

The PCSAs were taken directly from the morphological muscle parameters measured for the dissected specimen and a maximum muscle stress value of 35 N cm^−2^ was used to calculate the maximal isometric force of each muscle.^13^,^25^

The inertial parameters of the hand, i.e., the moments of inertia, the mass and the centre of mass position, were obtained using regression equations,^30^ employing the hand dimensions of the dissected specimen. The inertia matrix was calculated at the wrist joint centre using the Huygens-Steiner theorem. The position of the centre of mass was constrained to lie on the longitudinal axis of the MC3, defined by the location of the wrist and third metacarpophalangeal joint centres.

The resistive moment due to passive contributions was defined as a function of joint angle^28^

8 $$M_{\text{p}} = - k_{\text{p}} \cdot \theta + \frac{{M_{\text{p}}^{\hbox{max} } }}{{\exp \left( {\xi_{\text{p}} } \right)}}\left[ {\exp \left( {\frac{{\xi_{\text{p}} }}{{\theta_{\hbox{max} } }}\theta } \right) - 1} \right],$$

where *M*_p_^max^ is the maximal passive moment, *θ*_max_ the joint angle at which the maximal passive moment occurs, *k*_p_ the linear stiffness, *ξ*_p_ the shape parameter for the non-linear elastic component. Four relationships were defined, one for each direction of motion in the two degrees of freedom. The parameters of each of these four equations were manually adjusted (Table 1) to agree with wrist passive moment data measured by Delp *et al*.^11^

**Table 1:** Values used to define the relationships between passive moment at the wrist and joint angles. Definition of the parameters is provided in the text.

	Parameter values			
	*M*_p_^max^ (Nm)	*θ*_max_ (°)	*k* (Nm/°)	*ξ*
Extension	10	− 80	0.05	19
Flexion	− 10	90	0.3	12
Ulnar deviation	10	− 40	0.2	10
Radial deviation	− 10	30	0.2	7

#### Alternative Versions of the Model

To illustrate the effect of combining different datasets in the same musculoskeletal model, two alternative versions of the model were also tested, one where PCSAs were taken from Chao *et al*.,^7^ and another where moment arms were calculated directly from joint angles using polynomial regressions of Lemay and Crago.^15^

#### Model Evaluation and Simulations

The model was evaluated by comparing excursion and moment arm values with those reported from cadaver studies.^4^,^14^ The model was used to estimate muscle forces during a flexion–extension cycle of 1 s with the joint angle following a sine wave starting and finishing at 0° with maximal flexion and extension of 70°. To analyse the effect of the direction of gravity on the muscle forces, this motion was simulated with the forearm placed horizontally in two different positions—the ‘palm down’ position (gravity vector in the palmar direction) and the ‘thumb-up’ position (gravity vector in the ulnar direction).

## Results

### Anatomical Dataset

In total, the anatomical dataset includes 52 landmarks, 233 muscle points, 24 functional axes as well as the morphological parameters of 48 muscles and muscle bellies. For brevity, only the data used in the wrist musculoskeletal model are presented herein (Tables 2, 3, 4, 5, and 6). However, the entire dataset is accessible within the supplementary materials.

**Table 2:** Anthropometry of the specimen.

Measurement	Additional information	Value (mm)
Forearm length	Distance from lateral epicondyle to radial styloid	274
Wrist circumference	At the level of the wrist crease, just proximal to styloids	200
Hand circumference	At the level of metacarpophalangeal joints, fingers adducted	223
Hand width	Maximal distance along the line formed by the centre of the 2nd and 5th metacarpal heads, fingers adducted	87
Hand length	Measured in the palmar plane, from distal crease of the wrist to middle finger tip	203

**Table 3:** Location of digitized bony landmarks used in the musculoskeletal model of the wrist expressed in the ulna coordinate system^29^ in the reference posture.

Landmark	Definition	*X* (mm)	*Y* (mm)	*Z* (mm)
HE	Centre of the bony section where the humerus was cut	97.6	420.6	− 31.9
LE	Most lateral point of lateral epicondyle	0.0	274.4	− 34.0
ME	Most medial point of medial epicondyle	0.0	293.3	34.0
UO	Most posterior point of olecranon process	− 27.0	289.0	2.1
US	Most ulnar point of ulnar styloid	0.0	0.0	0.0
DRUJ	Most radial point on the dorsal aspect of the distal radio-ulnar joint	15.1	6.2	− 10.6
RS	Most radial point of radial styloid	62.4	8.2	2.4
MC3b	Most proximal point on the dorsal aspect of the 3rd metacarpal base	34.7	− 30.4	− 19.6
MC3h	Most distal point on the dorsal aspect of the 3rd metacarpal head	39.5	− 86.0	− 22.6

**Table 4:** Orientation of the functional axis and location of a point on the axis for flexion–extension (FE) and radial–ulnar deviation (RUD) of the wrist expressed in the ulna coordinate system^29^ in the reference posture.

Motion	Axis				Point			
	*X*	*Y*	*Z*	*δ* (*°*)	*X* (mm)	*Y* (mm)	*Z* (mm)	*Δ* (mm)
FE	− 0.971	− 0.227	− 0.082	*8.4*	43.229	− 0.712	− 2.087	*3.8*
RUD	0.030	0.155	0.987	*13.8*	38.585	− 11.269	− 3.927	*6.1*

The average variation in direction (*δ*) and position (*Δ*) of the instantaneous helical axes compared to the functional axis were calculated as in Veeger *et al*.^26^

**Table 5:** Location of muscle origin, insertion and via points for the flexor carpi ulnaris (FCU), flexor carpi radialis (FCR), palmaris longus (PL), extensor carpi ulnaris (ECU), extensor carpi radialis longus (ECRL), extensor carpi radialis brevis (ECRB) and abductor pollicis longus (APL) used in the musculoskeletal model of the wrist expressed in the ulna coordinate system^29^ in the reference posture.

Muscle	Point	Segment	*X* (mm)	*Y* (mm)	*Z* (mm)
FCU	O1	HUM	0.7	285.7	30.7
	I	MC3	19.2	− 16.9	19.8
FCR	O	HUM	7.7	298.7	28.1
	via	RAD	40.6	14.6	18.0
	I	MC3	52.5	− 33.5	− 2.8
PL	Op	HUM	9.4	306.1	27.2
	via2	MC3	35.3	− 30.7	17.3
	I2	MC3	44.1	− 85.9	− 0.7
ECU	Op*	HUM	− 8.2	281.0	− 25.3
	via1	RAD	5.9	26.8	− 3.5
	I	MC5	8.2	− 27.0	− 6.6
ECRL	Op	HUM	36.9	327.8	− 24.8
	via	RAD	54.2	27.8	− 0.9
	I	MC2	57.9	− 32.0	− 14.3
ECRB	Op	HUM	13.7	304.8	− 32.1
	via	RAD	46.9	23.9	− 3.4
	I	MC3	49.7	− 25.7	− 20.5
APL	Op	ULN	− 3.1	232.5	− 0.1
	via1	RAD	60.0	13.3	8.9
	I	MC1	79.5	− 19.7	4.3

“O”, “via” and “I” designate origin, *via* and insertion points, respectively. “p” designates the most proximal point of the origin or insertion area. * Designates the middle point of the area of the origin or insertion. When several origins, *via* or insertion points could be identified, a number was added at the end of the point name, e.g., O1. The point names in this table refer to those of the complete dataset, available as supplementary materials

**Table 6:** Morphological parameters of the flexor carpi ulnaris (FCU), flexor carpi radialis (FCR), palmaris longus (PL), extensor carpi ulnaris (ECU), extensor carpi radialis longus (ECRL), and extensor carpi radialis brevis (ECRB) and abductor pollicis longus (APL) in the musculoskeletal model of the wrist measured after fixation.

	Mass (g)	PCSA (cm^2^)	Pennation angle (°)	Belly length (mm)	Fibre length (mm)	Index of architecture	Tendon length (mm)
FCU	62.1	7.3	35	394	81	0.20	239
FCR	32.3	3.9	18	251	78	0.31	209
PL	12.0	1.5	22	206	76	0.37	239
ECU	42.4	8.9	35	255	45	0.18	249
ECRL	55.1	6.1	42	163	86	0.53	234
ECRB	41.9	6.2	33	182	64	0.35	217
APL	18.6	3.0	29	212	58	0.27	169

**Figure 3:**
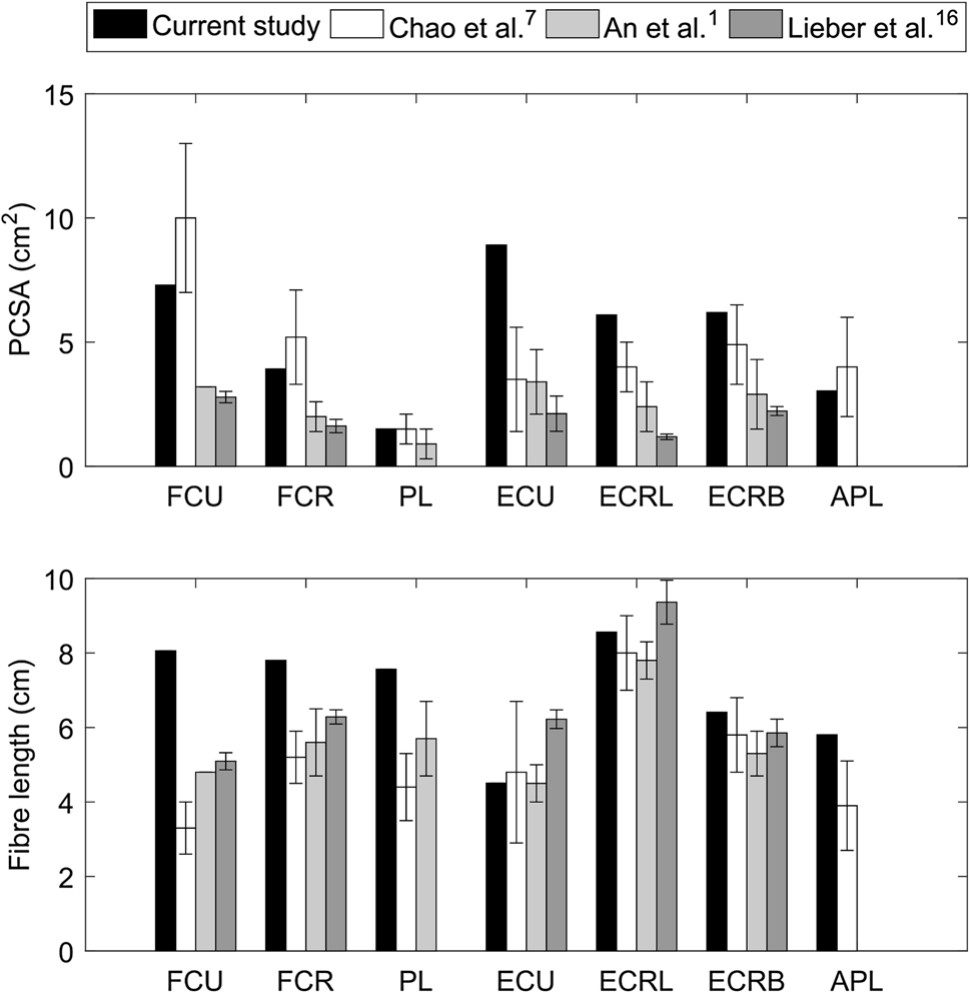
Physiological cross-sectional areas (PCSAs) and fibre lengths from the current study and as reported in the literature (mean ± SD) for the flexor carpi ulnaris (FCU), flexor carpi radialis (FCR), palmaris longus (PL), extensor carpi ulnaris (ECU), extensor carpi radialis longus (ECRL), extensor carpi radialis brevis (ECRB) and abductor pollicis longus (APL). From lightest to the darkest shade, the values were taken from Chao *et al*.,^7^ An *et al*.,^1^ and Lieber *et al*.^16^

The PCSAs and optimal fibre lengths measured for the wrist muscles are shown on Fig. 3, together with data from previous studies.^1^,^7^,^16^ PCSA values measured in the current study ranged from 1.5 cm^2^ for PL to 8.9 cm^2^ for ECU and were comparable with literature values. Only the PCSA of the extensors, i.e., ECU, ECRL and ECRB, were higher, up to 140% for ECU, than in previous studies. Fibre lengths ranged from 8.6 cm for ECRL to 4.5 cm for ECU and were also comparable with literature values. The fibre lengths of the flexors, i.e., FCU, FCR and PL, were higher, up to 60% for FCU, than in previous studies.

### Musculoskeletal Model of the Wrist

The parameters of the cylinders constraining the paths of the tendons are given in Table 7.

**Table 7:** Parameters of cylinders used to constraint the tendon paths in the musculoskeletal model for flexion (flex) and extension (ext) of the wrist for the flexor carpi ulnaris (FCU), flexor carpi radialis (FCR), palmaris longus (PL), extensor carpi ulnaris (ECU), extensor carpi radialis longus (ECRL), and extensor carpi radialis brevis (ECRB) and abductor pollicis longus (APL).

	Point			Axis			Radius
	X	Y	Z	X	Y	Z	
FCU							
Cylinder 1 (flex)	25.9	− 256.9	50.2	− 0.971	− 0.227	− 0.081	4.0
Cylinder 2 (ext)	30.3	− 273.2	33.3	− 0.971	− 0.227	− 0.081	12.0
FCR							
Cylinder 1 (flex)	28.8	− 269.1	50.8	− 0.971	− 0.227	− 0.081	2.5
Cylinder 2 (ext)	31.3	− 277.9	34.9	− 0.971	− 0.227	− 0.081	12.0
PL							
Cylinder 1 (flex)	25.3	− 254.7	55.9	− 0.971	− 0.227	− 0.081	5.0
Cylinder 2 (ext)	29.9	− 272.0	35.6	− 0.971	− 0.227	− 0.081	15.0
ECU							
Cylinder 1 (flex)	30.3	− 273.8	36.9	− 0.971	− 0.227	− 0.081	10.0
Cylinder 2 (ext)	29.5	− 269.3	25.7	− 0.971	− 0.227	− 0.081	1.5
ECRL							
Cylinder 1 (flex)	30.9	− 275.5	30.6	− 0.971	− 0.227	− 0.081	10.0
Cylinder 2 (ext)	29.6	− 269.2	20.6	− 0.971	− 0.227	− 0.081	1.5
ECRB							
Cylinder 1 (flex)	30.8	− 275.0	27.0	− 0.971	− 0.227	− 0.081	10.0
Cylinder 2 (ext)	29.0	− 266.2	18.7	− 0.971	− 0.227	− 0.081	1.5
APL							
Cylinder 1 (flex)	29.1	− 269.0	39.7	− 0.971	− 0.227	− 0.081	1.0
Cylinder 2 (ext)	30.7	− 275.1	34.0	− 0.971	− 0.227	− 0.081	5.0

The point coordinates and the radii are provided in mm

**Figure 4:**
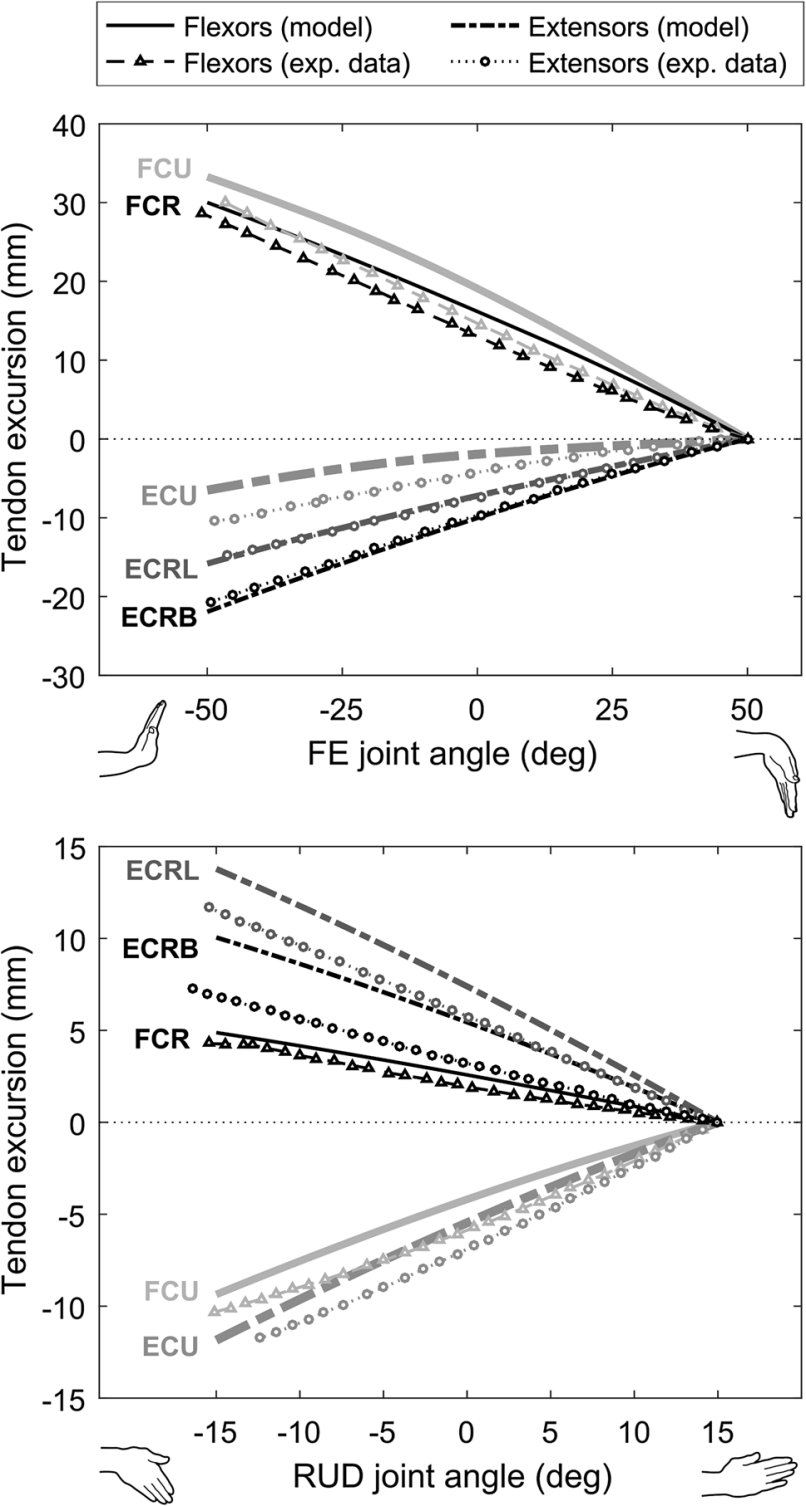
Comparison of tendon excursion-joint angle relationships with the experimental data reported in Horii *et al*.^14^ in flexion–extension (FE; upper panel) and radial–ulnar deviation (RUD; lower panel) for the flexor carpi ulnaris (FCU), flexor carpi radialis (FCR), palmaris longus (PL), extensor carpi ulnaris (ECU), extensor carpi radialis longus (ECRL), extensor carpi radialis brevis (ECRB) and abductor pollicis longus (APL).

The tendon excursion-joint angle relationships estimated with the model in both flexion–extension and radial–ulnar deviation are shown in Fig. 4, together with experimental measurements from a previous study.^14^ Over the 100° range of flexion–extension, the total excursions ranged, in absolute value, between 6.4 mm for ECU and 33.2 mm for FCU. The largest differences between model predictions and experimental values were observed for FCU (4.4 mm; + 24%) and ECU (4.09 mm; − 39%). Over the 30° of radial–ulnar deviation, the total excursions ranged, in absolute value, between 4.9 mm for FCR and 13.8 mm for ECRL. The greatest differences between model predictions and experimental values were observed for ECRB (3 mm; + 53%) and FCU (1.7 mm; − 23%).

**Figure 5:**
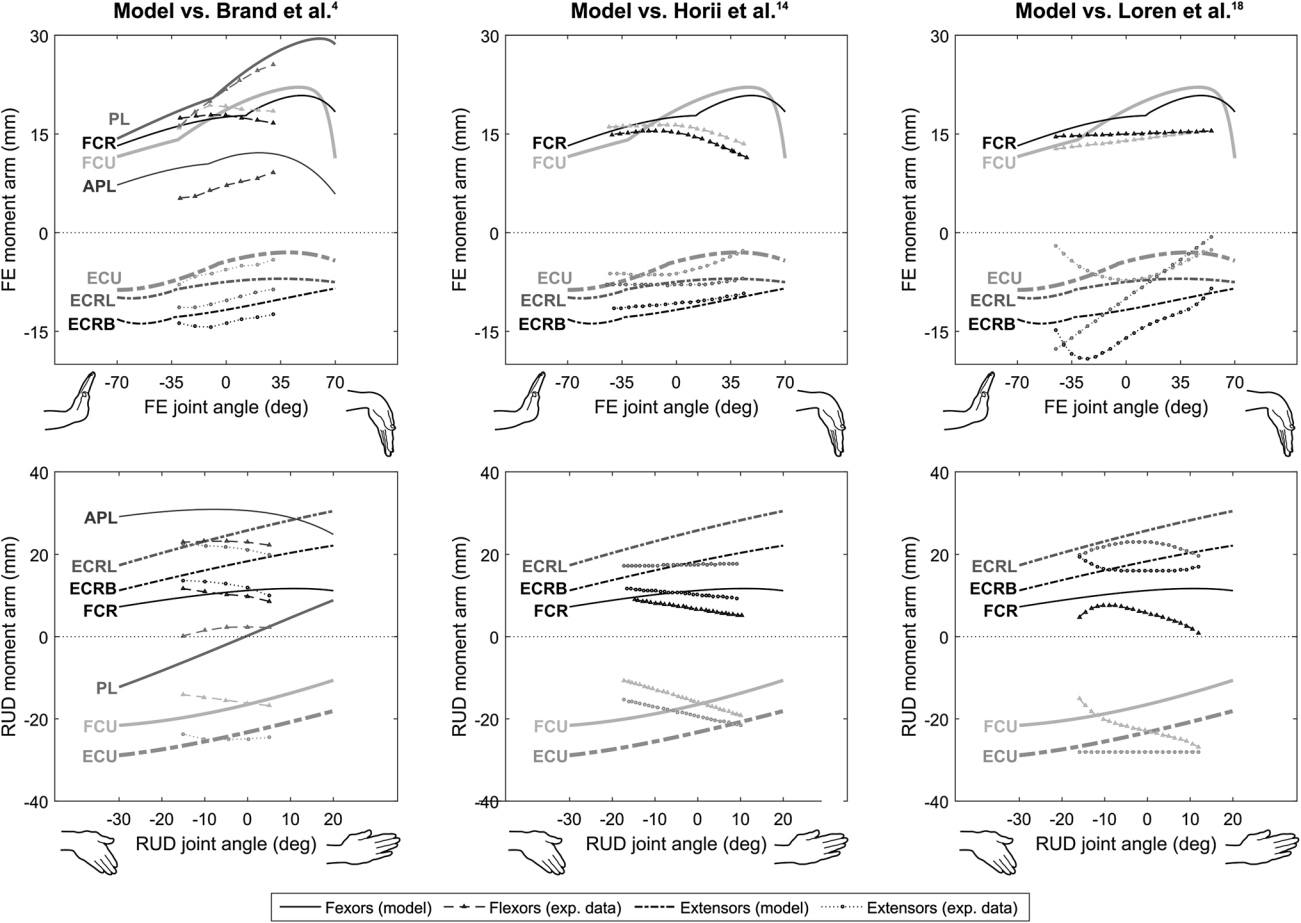
Comparison of moment arm-joint angle relationships with the experimental data reported in different studies for flexion–extension (FE; upper panels) and radial–ulnar deviation (RUD; lower panels) for the flexor carpi ulnaris (FCU), flexor carpi radialis (FCR), palmaris longus (PL), extensor carpi ulnaris (ECU), extensor carpi radialis longus (ECRL), extensor carpi radialis brevis (ECRB) and abductor pollicis longus (APL). Experimental data was taken from Brand and Hollister^4^ in left panels, Horii *et al*.^14^ in middle panels, Loren *et al*.^18^ in right panels.

The tendon moment arm-joint angle relationships obtained with the model are shown on Fig. 5, together with experimental data previously reported in the literature. The flexion–extension moment arms estimated by the model ranged, in absolute values, from 3.0 mm for ECU to 29.5 mm for PL. The greatest differences between the model and literature values were 4.7 mm (+ 85%) for APL when compared to Brand and Hollister,^4^ and 8.6 mm (+ 64%) and 6.3 mm (+ 40%) for FCU when compared to Horii *et al*.^14^ and Loren *et al*.,^18^ respectively. The moment arms in radial–ulnar deviation estimated by the model ranged, in absolute values, from 7.2 mm for FCR to 30.9 mm for APL. The largest differences between the model and literature values were 9.5 mm (+ 95%) and 10.8 mm (+ 115%) for ECRB when compared to Brand and Hollister^4^ and Horii *et al*.,^14^ respectively, and 13.6 mm (− 51%) for FCU when compared to Loren *et al*.^18^

**Figure 6:**
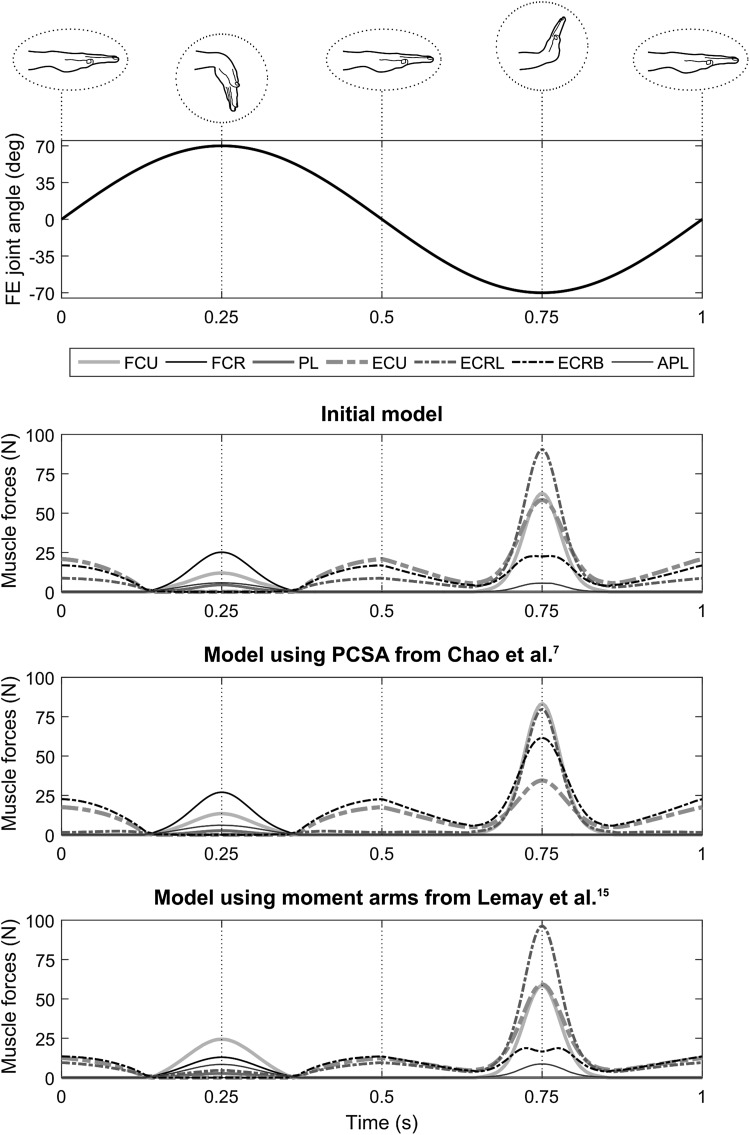
Muscle forces estimated with different versions of the model during the simulated flexion–extension (FE) cycle (upper panel) in the palm-down position for the flexor carpi ulnaris (FCU), flexor carpi radialis (FCR), palmaris longus (PL), extensor carpi ulnaris (ECU), extensor carpi radialis longus (ECRL), extensor carpi radialis brevis (ECRB) and abductor pollicis longus (APL). The last three lower panels present the results of, from top to bottom, the initial model, the model using physiological cross-sectional area (PCSA) values from Chao *et al*.^7^ and the model using moment arm-angle relationships from Lemay and Crago.^15^

**Figure 7:**
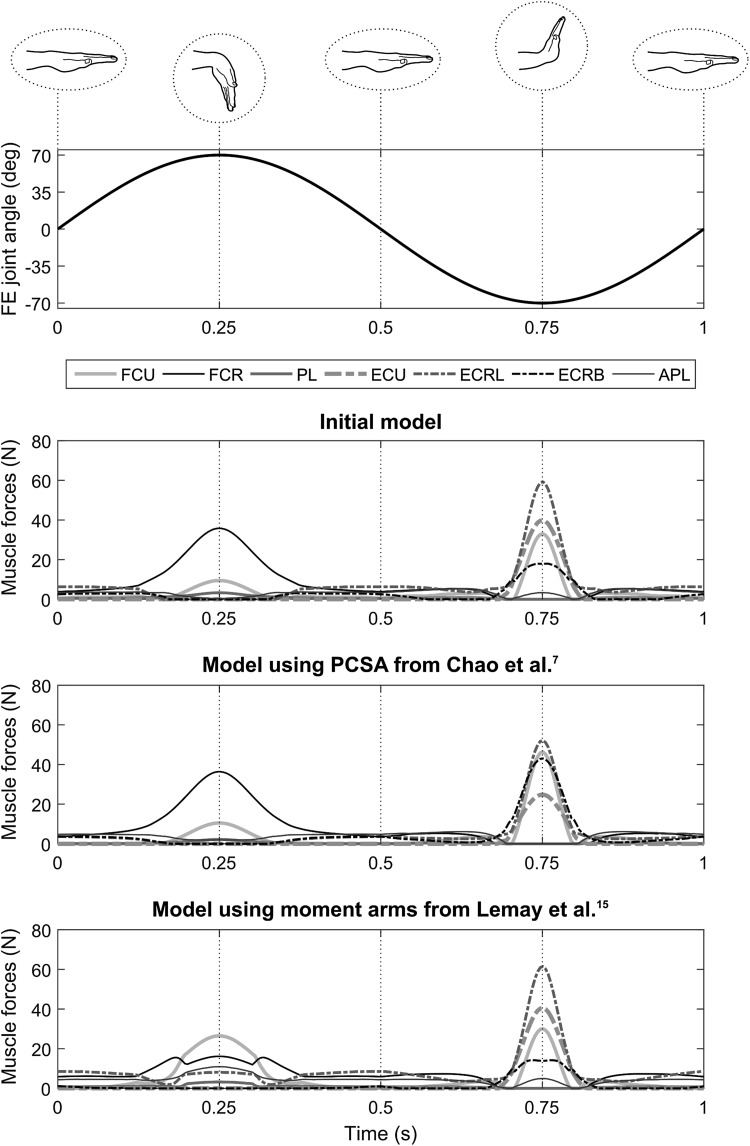
Muscle forces estimated with different versions of the model during the simulated flexion–extension (FE) cycle in the thumb-up position for the flexor carpi ulnaris (FCU), flexor carpi radialis (FCR), palmaris longus (PL), extensor carpi ulnaris (ECU), extensor carpi radialis longus (ECRL), extensor carpi radialis brevis (ECRB) and abductor pollicis longus (APL). The last three lower panels present the results of, from top to bottom, the initial model, the model using physiological cross-sectional area (PCSA) values from Chao *et al*.^7^ and the model using moment arm-angle relationships from Lemay and Crago.^15^

The muscle forces estimated by the initial musculoskeletal model during the simulated flexion–extension motion in the two tested positions showed comparable trends, but resulted in different muscle load sharing and force intensities (Figs. 6 and 7). In the palm-down position, the flexors were active during less than half of the cycle, and for less than a quarter of the cycle for PL and FCR. They reached their maximal force, up to 25.2 N for FCR, at 0.25 s, i.e., when the wrist was at 70° of flexion, with the exception of FCU, which reached its maximum force of 62.6 N at 0.75 s. The extensors were active during more than three quarters of the cycle and were all inactive in the interval between 0.2 and 0.3 s; their force was maximal, up to 90.0 N for ECRL, at 0.75 s, i.e., when the wrist was at 70° of extension. In the thumb-up position, some flexors were active during more than three quarters of the cycle. Their forces were maximal at 0.25 s for PL and FCR (3.3 and 35.8 N, respectively), at 0.63 s for APL (5.4 N), and at 0.75 s for FCU (75.1 N). During this same motion, ECRB and ECRL were active for more than 80% of the cycle, whereas ECU was active for less than half of the motion. The forces of the three extensors were all maximal at 0.75 s, with up to 59.3 N for ECRL.

Compared to the initial model, the two alternative versions of the model resulted in different patterns of muscle forces and muscle load sharing. The use of PCSAs from the literature particularly modified the load sharing between the extensors. During the palm-down motion, ECRL and ECU forces were reduced by up to 82 and 40%, respectively, whereas ECRB force was increased by up to 179% at 0.75 s of the cycle. During the thumb-up motion, similar modifications were observed; ECRL and ECU were decreased by up to 52 and 38%, respectively, and ECRB was increased by up to 141% at 0.75 s. Using the PCSAs from the literature, FCU was the only flexor for which muscle force was affected, with increases of up to 33% and 40% in both the palm-down and thumb-up, respectively. Using moment arms from the literature, the load sharing between both flexors and extensors was modified. In both motions, this model resulted in modified muscle load sharing of the flexors on the interval between 0.15 and 0.35 s with the FCU force being increased by up to 182% and the FCR force being decreased by up to 55%. During the palm-down motion, the extensors were particularly affected in the interval between 0.35 and 0.65 s of the cycle, with ECU and ECRB forces being reduced by up to 40 and 20%. During the thumb-up motion, although muscle force intensities were not modified greatly, ECRL was active throughout the trial, including between 0.20 and 0.30 s, which was not the case with the initial model.

## Discussion

A large anatomical dataset was collected to provide a quantified representation of the musculoskeletal system from the elbow to the tip of the five fingers for musculoskeletal modelling of the hand and the wrist. Motion capture, muscle fixation and clinical imaging were combined to characterize the musculoskeletal geometry, the muscle morphology, and the bone surfaces of a single specimen. The collection protocol followed the one described in Mirakhorlo *et al*.^19^ who completed a similar study and also provided an anatomical dataset. Compared to this previous study, the specimen dissected here was male, younger, and larger in terms of anthropometry. In addition, the present dataset also provides clinical imaging (both CT and MRI) which was not available with the previous study. Finally, simple geometrical shapes were proposed here to constrain the paths of tendons at the wrist, which was not available previously.

A musculoskeletal model of the wrist, with two degrees of freedom, actuated by seven muscles, was developed to estimate muscle forces and demonstrate the potential of the collected dataset. The comparison of the model’s outputs with direct cadaveric measurements showed that using two cylinders to constrain the path of each tendon was sufficient to obtain functionally realistic results. The tendon excursions in the model, calculated using the two-cylinder approach (Fig. 2b),^3^ were in good agreement with cadaveric experimental curves^14^ (Fig. 4). The comparison of moment arms in the model with those measured functionally on cadavers showed that the distribution across muscles were in relatively good agreement but that the shapes of the curves occasionally differed (Fig. 5). The general discrepancies between modelled and experimental values in radial–ulnar deviation can be explained by the fact that the two-cylinder approach predominantly constrains the tendon paths during flexion–extension. Nevertheless, the curves taken from cadaveric studies are mean curves and therefore are representative of trends in a population, whereas the model represents a single specimen. Furthermore, none of these studies provided a measure of the inter-subject variations; therefore, although the curves of the model diverged from those of the literature, they might remain within a physiological range of the mean curves. Finally, the anthropometry of the specimens in those previous studies was not provided; therefore, it is not possible to normalize the moment arms and remove any size effects in the comparisons.

The muscle forces estimated by the model during the two simulated motions were consistent with the mechanical constraints of the motion and included estimations of co-contraction (Figs. 6 and 7). The muscles that were most activated throughout the flexion–extension cycle were the extensors in the palm-down position and the radial deviators in the thumb-up position. This is explained by the fact that these muscle groups were the ones balancing the action of gravity on the hand, which resulted in a flexion moment in the palm-down position and an ulnar moment in the thumb-up position. In both positions, most of the flexors and the extensors reached their maximal muscle force at the times corresponding to the maximal flexion angle and the maximal extension angle, respectively. This is consistent with the development of contributions from passive structures, which created a resistive moment that increased exponentially when approaching the limits of the range of motion and ultimately outweighed the hand acceleration moment. More interestingly, in the interval where extension angle was maximal, i.e., around 0.75 s, the model estimated a co-contraction of both FCU and APL muscles. This co-contraction was due to the constraint on the direction of the joint reaction force, preventing unrealistically high shear components. This result is encouraging because the estimation of co-contraction using musculoskeletal modelling is difficult and often requires the use of electromyography to guide the choice of a solution in the muscle load sharing problem.^12^ However, in other parts of the trial, only one muscle group is involved, e.g., extensors in the palm-down position, which might seem unrealistic. Unfortunately, because of the lack of data regarding muscular activity and loading of the forearm muscles during simple wrist tasks, it is difficult to fully validate the muscle force and co-contraction levels estimated by the model. However, the tendon excursion and moment arms estimated by the model were consistent with experimental data giving confidence that the model is realistic.

The two alternative versions of the model that combined data collected in the present study with average data previously reported in the literature markedly modified the muscle force levels and the muscle load sharing predicted by the model (Figs. 6 and 7). Using average PCSAs particularly modified the load sharing between the extensors. This could be expected since the PCSAs of extensors were markedly larger in the current study whereas those of flexors were closer to literature data (Fig. 3). The muscle stress criteria (Eq. 2) chooses a muscle load sharing solution to optimise the contribution of each muscle according to its PCSA, reflecting its maximal force capacity. Therefore, if a given muscle is assigned a larger PCSA, the optimisation will try to reduce the contribution of that muscle. In general, the variations in and distribution of these parameters, are probably due to differences in terms of age or anthropometry between the specimen dissected in this study and the populations considered in the previous studies. Using polynomial regressions^15^ to calculate moment arms affected the flexors more strongly, for example the FCU force increased by up to 182%, with some variations in the load sharing of the extensors. As with PCSAs, the distribution of moment arms across the muscles directly impacts muscle load sharing since it represents how much each muscle can contribute to balance the resultant moment at the joint (Eq. 3). Although the moment arms in the initial model were globally close to those of Brand and Hollister^4^ (Fig. 5), this modification influenced the muscle force estimations. More importantly, the regression equations taken from the literature assumed the moment arm about an axis varies only with the joint angle about that same axis and thus neglected the fact that each moment arm component depends on the posture of the joint, which is described by two joint angles for the wrist. Using 3D coordinates of the tendon *via* points in combination with geometrical constraints, as in the initial model, represents a more realistic representation of the joint biomechanics. The two alternative versions of the model demonstrated how much the combination of different datasets within a same model can markedly influence the predicted muscle load sharing, and confirmed the importance of using the same source of data for musculoskeletal geometry and muscle morphology.

Several limitations should be considered regarding the results of the present study. Regarding the dataset, the parameters provided herein describe the musculoskeletal geometry and muscle morphology of a single specimen and might be inadequate to represent the anatomy of some subjects. Further studies should therefore investigate how to personalize these parameters so that the dataset can be used to model participants presenting different anthropometries or different muscle force capacities. Furthermore, the bone surfaces obtained from the clinical imaging were manually registered to match the data measured with motion capture during the dissection. Automated registration may have been possible if the clusters were affixed to the specimen prior to imaging, but this would have required dissection prior to the scans, which would have complicated the scanning protocol. In addition, a greater number of scans would have been required, as clusters could not be placed on all the fingers at once. More generally, despite the quality and quantity of data provided in the present study, the users of this dataset should bear in mind that it faces inherent limitations related to all cadaveric experiments. For instance, the parameters provided might vary with joint motions or muscle contraction levels, e.g., muscle morphological parameters and positions of the tendons relative to the bones. Considering the high number of muscles tested, investigating such variations would have drastically increased the time of the dissection, detrimentally affecting the integrity of the tissues. However, we are confident that the quantitative dataset provided in this study represents a reliable reference to design models of the musculoskeletal system of the hand and the forearm

The action of hand extrinsic muscles at the wrist was not included in the model. Although this might have influenced the muscle load sharing predicted by the model, adding these muscles would have necessitated the inclusion of the degrees of freedom at the finger joints, as well as the actions of the intrinsic hand muscles at those joints. However, our intentions were to demonstrate the potential of the dataset using a relatively simple musculoskeletal model of the wrist, and to illustrate the consequences of combining datasets.

In conclusion, the anatomical dataset provided here will enable the development of a complex model of the musculoskeletal system of the hand and wrist, from the elbow to the tips of the fingers. The wrist musculoskeletal model presented herein demonstrated that the dataset can provide physiologically realistic estimations of tendon excursions and moment arms and can be further used to predict muscle forces during simple motions. This large dataset can be used to develop and improve musculoskeletal models of the hand, and therefore, ameliorate the predictions of the geometrical and biomechanical behaviour of the hand structure. This could facilitate the quantitative assessment of hand internal biomechanics and therefore, improve ergonomics, rehabilitation and the prevention of musculoskeletal disorders.

## Electronic Supplementary Material

ESM (PDF 509 KB)

## Abbreviations

APL: Abductor pollicis longus
CT: Computed tomography
ECRB: Extensor carpi radialis brevis
ECRL: Extensor carpi radialis longus
ECU: Extensor carpi ulnaris
FE: Flexion–extension
ISB: International Society of Biomechanics
FCR: Flexor carpi radialis
FCU: Flexor carpi ulnaris
MC3: Third metacarpal
MRI: Magnetic resonance imaging
PCSA: Physiological cross-sectional area
PL: Palmaris longus
RUD: Radial–ulnar deviation

## List of symbols

*m*_*m*_: Mass of muscle *m*
*ρ*_*m*_: Density of muscle *m*
*l*_*f*_^0^: Optimal fibre length
*f*_*m*_: Muscle force of muscle *m*
[**I**_*h*_]: Inertia matrix of the hand at the wrist joint centre
${{\vec{\ddot{\varvec{\theta}}}}}\left( t \right)$: Angular acceleration of the hand centre of mass
${\vec{\mathbf{M}}}_{p}$: Moment due to passive constraints in the wrist joint
${\vec{\mathbf{M}}}_{e}$: Moment due to the external forces applied to the hand
${\vec{\mathbf{r}}}_{m}$: Moment arm vector of muscle *m*
*σ*_max_: Maximum muscle stress
${\vec{\mathbf{R}}}_{j}$: Joint reaction force at the wrist
${\vec{\mathbf{x}}}_{\text{radius}}$: Antero-posterior axis of the radius
${\vec{\mathbf{y}}}_{\text{radius}}$: Longitudinal axis of the radius
${\vec{\mathbf{z}}}_{\text{radius}}$: Medio-lateral axis of the radius
*α*: Angle between antero-posterior component of joint reaction force and longitudinal axis of the radius
*β*: Angle between medio-lateral component of joint reaction force and longitudinal axis of the radius
*m*_*h*_: Mass of the hand
${\vec{\mathbf{a}}}_{h}$: Acceleration of the hand centre of mass
${\vec{\mathbf{F}}}_{e}$: Resultant force due to external forces applied to the hand
${\vec{\mathbf{u}}}_{m}$: The unit vector describing the line of action of muscle *m*
*k*_*p*_: Linear stiffness of passive structures at the wrist
$\xi_{p}$: Shape parameter for modelling non-linear elastic stiffness of passive structures at the wrist
