# Supplementary material for: Importance of Consistent Datasets in Musculoskeletal Modelling: A Study of the Hand and Wrist
Source: Ann Biomed Eng. 2017 Oct 2;46(1):71–85. doi: 10.1007/s10439-017-1936-z (PMC5754461; doi:10.1007/s10439-017-1936-z)
Supplement: Supplementary file 1 — ESM (PDF 509 KB) [file 10439_2017_1936_MOESM1_ESM.pdf]

Supplementary Material for 'Importance of consistent datasets in musculoskeletal modelling: a study of the hand and wrist' (Goislard de Monsabert et al.)

This document contains the complete anatomical dataset measured on the specimen as described in the article.

Table S1 contains the anthropometry of the specimen

Table S2a-f contains the 3D coordinates of digitized bony landmarks

Table S3a-f contains the 3D orientation and position of the functional joint rotation axes

Table S4a-g contains the 3D coordinates of via points describing the muscle/tendon path

Table S5a-b contains the muscle morphological parameters

Table S6 contains the parameters of the cylinders constraining tendon paths at the wrist.

Note: all 3D coordinates presented in Table S2a-f, S3a-f, S4a-g and S6 are expressed in the ulna coordinate system as described in the ISB recommendations[1]:

- The Y axis is the unit vector describing the line between the ulnar styloid (US) and the mid-point between medial (ME) and lateral (LE) epicondyles, pointing proximally.
- The X axis is the unit vector perpendicular to the plane formed by US, ME and LE, pointing anteriorly.
- The Z axis is perpendicular to X and Y, pointing in the ulnar direction.
- The origin is at US

[1] Wu, G., F. C. T. van der Helm, H. E. J. D. Veeger, M. Makhsous, P. Van Roy, C. Anglin, J. Nagels, A. R. Karduna, K. McQuade, X. Wang, F. W. Werner, and B. Buchholz. ISB recommendation on definitions of joint coordinate systems of various joints for the reporting of human joint motion--Part II: shoulder, elbow, wrist and hand. *J. Biomech.* 38:981–992, 2005.

Table S1 – Anthropometry of the specimen.

Measurement	Additional information	Value (mm)
Forearm length	Distance from lateral epicondyle to radial styloid	274
Hand length	Measured in the palm plane, from distal crease of the wrist to middle finger tip	203
Middle finger length	Measured in the palm plane, from most anterior point of 3rd metacarpal head to middle finger tip	102
Elbow width	Maximal distance along the line formed by the epicondyles	121
Wrist width	Maximal distance along the line formed by the styloids	70
Hand width	Maximal distance along the line formed by the centre of the 2nd and 5th metacarpal heads, fingers adducted	87
Elbow circumference	At the level of elbow crease and passing by epicondyles	324
Forearm circumference	Maximum circumference of forearm measured in the transverse plane	328
Wrist circumference	At the level of the wrist crease, just proximal to styloids	200
Hand circumference	At the level of metacarpophalangeal joints, finger adducted	223

Table S2a – Location of arm and forearm bony landmarks in the neutral reference posture expressed in the ulna coordinate system.

Landmark	Definition	X (mm)	Y (mm)	Z (mm)
HE	Centre of the bony section where the humerus was cut	97.6	420.6	-31.9
LE	Most lateral point of lateral epicondyle	0.0	274.4	-34.0
ME	Most medial point of medial epicondyle	0.0	293.3	34.0
UO	Most posterior point of olecranon process	-27.0	289.0	2.1
US	Most ulnar point of ulnar styloid	0.0	0.0	0.0
DRUJ	Most radial point on the dorsal aspect of the distal radio-ulnar joint	15.1	6.2	-10.6
RS	Most radial point of radial styloid	62.4	8.2	2.4

Table S2b – Location of thumb bony landmarks in the neutral reference posture expressed in the ulna coordinate system.

Landmark	Definition	X (mm)	Y (mm)	Z (mm)
MC1b	Most proximal point on the dorsal aspect of the 1st metacarpal base	73.2	-19.6	4.3
MC1h	Most distal point on the dorsal aspect of the 1st metacarpal head	92.6	-42.8	6.8
MCP1u	Most ulnar point of the 1st metacarpal head	89.3	-54.2	-1.8
MCP1r	Most radial point of the 1st metacarpal head	92.4	-48.0	16.0
PP1h	Most distal point on the dorsal aspect of the thumb proximal phalanx head	119.8	-79.0	9.0
IP1u	Most ulnar point of the thumb proximal phalanx head	113.7	-88.8	1.3
IP1r	Most radial point of the thumb proximal phalanx head	114.4	-83.0	20.5
DP1h	Most distal point of the thumb distal phalanx, just below nail	132.8	-110.8	14.8

Table S2c – Location of index finger bony landmarks in the neutral reference posture expressed in the ulna coordinate system.

Landmark	Definition	X (mm)	Y (mm)	Z (mm)
MC2b	Most proximal point on the dorsal aspect of the 2nd metacarpal base	46.1	-26.9	-24.2
MC2h	Most distal point on the dorsal aspect of the 2nd metacarpal head	64.3	-81.4	-24.3
PP2h	Most distal point on the dorsal aspect of the index finger proximal phalanx head	75.6	-127.9	-24.7
PIP2u	Most ulnar point of the index finger proximal phalanx head	70.3	-133.1	-16.6
PIP2r	Most radial point of the index finger proximal phalanx head	87.6	-126.6	-17.2
MP2h	Most distal point on the dorsal aspect of the index finger proximal phalanx head	78.6	-161.2	-18.9
DIP2u	Most ulnar point of the index finger proximal phalanx head	73.3	-161.5	-12.9
DIP2r	Most radial point of the index finger proximal phalanx head	89.2	-158.7	-15.9
DP2h	Most distal point of the index finger distal phalanx, just below nail	82.7	-183.1	-18.3

Table S2d – Location of middle finger bony landmarks in the neutral reference posture expressed in the ulna coordinate system.

Landmark	Definition	X (mm)	Y (mm)	Z (mm)
MC3b	Most proximal point on the dorsal aspect of the 3rd metacarpal base	34.7	-30.4	-19.6
MC3h	Most distal point on the dorsal aspect of the 3rd metacarpal head	39.5	-86.0	-22.6
PP3h	Most distal point on the dorsal aspect of the middle finger proximal phalanx head	49.0	-142.3	-20.4
PIP3u	Most ulnar point of the middle finger proximal phalanx head	40.7	-139.4	-13.2
PIP3r	Most radial point of the middle finger proximal phalanx head	62.3	-137.6	-15.1
MP3h	Most distal point on the dorsal aspect of the middle finger proximal phalanx head	52.9	-169.9	-20.9
DIP3u	Most ulnar point of the middle finger proximal phalanx head	47.1	-173.8	-8.7
DIP3r	Most radial point of the middle finger proximal phalanx head	63.6	-176.4	-14.2
DP3h	Most distal point of the middle finger distal phalanx, just below nail	60.4	-197.9	-20.7

Table S2e – Location of ring finger bony landmarks in the neutral reference posture expressed in the ulna coordinate system.

Landmark	Definition	X (mm)	Y (mm)	Z (mm)
MC4b	Most proximal point on the dorsal aspect of the 4th metacarpal base	26.7	-26.5	-15.5
MC4h	Most distal point on the dorsal aspect of the 4th metacarpal head	17.3	-76.6	-11.5
PP4h	Most distal point on the dorsal aspect of the ring finger proximal phalanx head	14.2	-125.5	-14.7
PIP4u	Most ulnar point of the ring finger proximal phalanx head	8.9	-126.3	-1.6
PIP4r	Most radial point of the ring finger proximal phalanx head	25.7	-127.7	-10.6
MP4h	Most distal point on the dorsal aspect of the ring finger proximal phalanx head	10.3	-159.3	-13.7
DIP4u	Most ulnar point of the ring finger proximal phalanx head	5.3	-157.4	-4.3
DIP4r	Most radial point of the ring finger proximal phalanx head	16.6	-159.1	-10.8
DP4h	Most distal point of the ring finger distal phalanx, just below nail	6.5	-184.6	-15.8

Table S2f – Location of little finger bony landmarks in the neutral reference posture expressed in the ulna coordinate system.

Landmark	Definition	X (mm)	Y (mm)	Z (mm)
MC5b	Most proximal point on the dorsal aspect of the 5th metacarpal base	11.0	-24.8	-9.3
MC5h	Most distal point on the dorsal aspect of the 5th metacarpal head	6.1	-71.7	-3.2
PP5h	Most distal point on the dorsal aspect of the little finger proximal phalanx head	-0.8	-112.0	-5.4
PIP5u	Most ulnar point of the little finger proximal phalanx head	-3.8	-114.5	4.4
PIP5r	Most radial point of the little finger proximal phalanx head	6.8	-113.8	-4.5
MP5h	Most distal point on the dorsal aspect of the little finger proximal phalanx head	-1.9	-134.8	-1.3
DIP5u	Most ulnar point of the little finger proximal phalanx head	-4.5	-132.6	5.3
DIP5r	Most radial point of the little finger proximal phalanx head	6.1	-133.4	1.3
DP5h	Most distal point of the little finger distal phalanx, just below nail	2.3	-158.1	0.9

Table S3a – Orientation of the functional axis and location of a point on the axis for each degree of freedom of the arm and forearm joints in the neutral reference posture expressed in the ulna coordinate system. The average variation in direction (δ) and position (Δ) of the instantaneous helical axes compared to the functional axis were calculated as in Veeger et al.[2]

	Axis				Point			
Joint	X	Y	Z	*δ (°)*	X (mm)	Y (mm)	Z (mm)	*Δ (mm)*
Elbow f-e	-0.045	0.148	0.988	*14.9*	13.498	259.631	-85.121	*13.9*
Forearm f-e	-0.082	0.982	-0.172	*15.6*	16.217	104.318	-2.197	*12.9*
Wrist f-e	-0.971	-0.227	-0.082	*8.4*	43.229	-0.712	-2.087	*3.8*
Wrist a-a	0.030	0.155	0.987	*13.8*	38.585	-11.269	-3.927	*6.1*

Table S3b – Orientation of the functional axis and location of a point on the axis for each degree of freedom of the thumb in the neutral reference posture expressed in the ulna coordinate system. The average variation in direction (δ) and position (Δ) of the instantaneous helical axes compared to the functional axis were calculated as in Veeger et al.[3]

	Axis				Point			
Joint	X	Y	Z	*δ (°)*	X (mm)	Y (mm)	Z (mm)	*Δ (mm)*
CMC1 f-e	-0.018	-0.931	-0.364	*33.6*	63.295	-34.518	-7.088	*7.2*
CMC1 a-a	0.896	0.235	-0.377	*47.0*	82.077	-28.811	-4.837	*14.1*
MCP1 f-e	-0.356	-0.482	-0.801	*21.4*	91.554	-64.140	3.117	*11.5*
IP1 f-e	-0.197	-0.290	-0.936	*22.4*	111.414	-95.813	-15.995	*7.2*

[2] Veeger, H. E. J., B. Yu, K.-N. An, and R. H. Rozendal. Parameters for modeling the upper extremity. *J. Biomech*. 30:647–652, 1997.

[3] ibid

Table S3c – Orientation of the functional axis and location of a point on the axis for each degree of freedom of the index finger in the neutral reference posture expressed in the ulna coordinate system. The average variation in direction (δ) and position (Δ) of the instantaneous helical axes compared to the functional axis were calculated as in Veeger et al.[4]

	Axis				Point			
Joint	X	Y	Z	*δ (°)*	X (mm)	Y (mm)	Z (mm)	*Δ (mm)*
MCP2 f-e	-0.929	-0.185	0.321	*18.3*	71.674	-87.610	-17.488	*5.6*
MCP2 a-a	0.234	0.154	0.960	*13.3*	68.471	-87.639	-14.211	*4.2*
PIP2 f-e	-0.982	-0.134	0.133	*29.1*	64.953	-135.492	-12.956	*9.4*
DIP2 f-e	-0.924	-0.125	0.362	*22.0*	76.188	-161.367	-16.307	*13.4*

Table S3d – Orientation of the functional axis and location of a point on the axis for each degree of freedom of the middle finger in the neutral reference posture expressed in the ulna coordinate system. The average variation in direction (δ) and position (Δ) of the instantaneous helical axes compared to the functional axis were calculated as in Veeger et al.[5]

	Axis				Point			
Joint	X	Y	Z	*δ (°)*	X (mm)	Y (mm)	Z (mm)	*Δ (mm)*
MCP3 f-e	-0.932	0.137	0.337	*3.9*	32.900	-88.269	-9.424	*1.0*
MCP3 a-a	0.257	-0.233	0.938	*5.9*	42.276	-88.635	-13.560	*1.3*
PIP3 f-e	-0.980	-0.088	0.181	*7.4*	44.415	-140.812	-13.250	*3.1*
DIP3 f-e	-0.923	-0.058	0.381	*13.3*	53.651	-174.594	-18.298	*4.0*

[4] ibid
[5] ibid

Table S3e – Orientation of the functional axis and location of a point on the axis for each degree of freedom of the middle finger in the neutral reference posture expressed in the ulna coordinate system. The average variation in direction (δ) and position (Δ) of the instantaneous helical axes compared to the functional axis were calculated as in Veeger et al.[6]

	Axis				Point			
Joint	X	Y	Z	*δ (°)*	X (mm)	Y (mm)	Z (mm)	*Δ (mm)*
MCP4 f-e	-0.884	0.050	0.465	*4.4*	25.619	-81.791	-4.356	*1.1*
MCP4 a-a	0.602	0.017	0.798	*6.8*	18.649	-85.173	-9.535	*1.2*
PIP4 f-e	-0.901	0.227	0.369	*7.5*	11.583	-126.928	-9.053	*2.5*
DIP4 f-e	-0.905	0.078	0.418	*24.6*	13.240	-160.772	-12.181	*6.8*

Table S3f – Orientation of the functional axis and location of a point on the axis for each degree of freedom of the middle finger in the neutral reference posture expressed in the ulna coordinate system. The average variation in direction (δ) and position (Δ) of the instantaneous helical axes compared to the functional axis were calculated as in Veeger et al.[7]

	Axis				Point			
Joint	X	Y	Z	*δ (°)*	X (mm)	Y (mm)	Z (mm)	*Δ (mm)*
MCP5 f-e	-0.928	0.228	0.294	*7.1*	-1.732	-75.912	4.286	*1.3*
MCP5 a-a	0.389	-0.482	0.785	*4.2*	2.748	-72.907	-9.168	*0.7*
PIP5 f-e	-0.861	0.119	0.494	*6.9*	2.405	-113.490	-1.615	*4.6*
DIP5 f-e	-0.909	-0.002	0.418	*17.5*	1.694	-136.401	-0.045	*7.9*

[6] ibid
[7] ibid

Table S4a – Location of origin, insertion and via points for arm and forearm supinator and pronator muscles in the neutral reference posture expressed in the ulna coordinate system.

Muscle	Point	Segment	X (mm)	Y (mm)	Z (mm)
BIC	via	HUM	55.5	289.8	-9.3
	I1r	ULN	37.5	265.3	7.2
	I1u	ULN	45.2	249.2	-9.9
	I2	RAD	26.9	227.5	-19.0
BRA	via	HUM	34.2	267.6	-1.7
	I	ULN	15.3	257.1	-16.3
	Op	HUM	59.1	377.8	-31.3
	Od	HUM	35.3	334.0	-26.0
	I	RAD	58.3	8.9	14.6
TRI	via	HUM	9.9	322.3	5.1
	I*	ULN	-7.9	300.1	-5.6
SUP	O*	ULN	-4.7	223.6	-23.7
	I*	RAD	10.6	205.7	-9.0
PRT	Op	HUM	14.8	307.7	14.5
	Od	HUM	7.1	301.9	27.8
	Ip	RAD	26.3	146.6	0.5
	Id	RAD	35.3	119.7	0.2
PRQ	O*	ULN	14.6	58.8	10.3
	I*	RAD	42.4	39.1	7.8

“O”, “via” and “I” designate origin, via and insertion points, respectively. “p”, “d”, ”u” and “r” designate the most proximal, distal, ulnar or radial point of the origin or insertion area, respectively. * designate the middle point of the area of origin or insertion. Extrinsic extensors have no insertion point because they insert on the extensor mechanisms on the dorsal aspect of the phalanges so that the last via point for each muscle represent the point connecting to the extensor mechanism.

Table S4b – Location of origin, insertion and via points for wrist prime movers in the neutral reference posture expressed in the ulna coordinate system.

Muscle	Point	Segment	X (mm)	Y (mm)	Z (mm)
PL	Op	HUM	9.4	306.1	27.2
	Od	HUM	5.1	299.6	32.4
	via1	RAD	39.9	-2.5	20.9
	via2	MC3	35.3	-30.7	17.3
	I1	MC2	63.4	-88.5	-7.5
	I2	MC3	44.1	-85.9	-0.7
	I3	MC4	25.9	-80.3	5.1
	I4	MC5	15.2	-70.8	3.6
FCU	O1	HUM	0.7	285.7	30.7
	O2 *	ULN	-10.1	173.6	-3.6
	via	RAD	12.7	18.1	16.5
	I	MC3	19.2	-16.9	19.8
FCR	O	HUM	7.7	298.7	28.1
	via	RAD	40.6	14.6	18.0
	I	MC3	52.5	-33.5	-2.8
ECU	Op*	HUM	-8.2	281.0	-25.3
	Od*	ULN	-12.7	261.7	-15.8
	via1	RAD	5.9	26.8	-3.5
	via2	MC3	14.1	-6.9	-0.2
	I	MC5	8.2	-27.0	-6.6
ECRL	Op	HUM	36.9	327.8	-24.8
	Od	HUM	19.8	294.6	-23.0
	via	RAD	54.2	27.8	-0.9
	I	MC2	57.9	-32.0	-14.3
ECRB	Op	HUM	13.7	304.8	-32.1
	Od	RAD	2.6	283.6	-2.6
	via	RAD	46.9	23.9	-3.4
	I	MC3	49.7	-25.7	-20.5

“O”, “via” and “I” designate origin, via and insertion points, respectively. “p”, “d”, ”u” and “r” designate the most proximal, distal, ulnar or radial point of the origin or insertion area, respectively. * designate the middle point of the area of origin or insertion. Extrinsic extensors have no insertion point because they insert on the extensor mechanisms on the dorsal aspect of the phalanges so that the last via point for each muscle represent the point connecting to the extensor mechanism.

Table S4c – Location of origin, insertion and via points for thumb muscles in the neutral reference posture expressed in the ulna coordinate system.

Muscle	Point	Segment	X (mm)	Y (mm)	Z (mm)
FPL	O*	RAD	35.5	133.2	-2.2
	via1	RAD	34.7	4.4	11.9
	via2	MC3	46.3	-28.6	6.7
	via3	MC1	87.5	-57.6	11.9
	via4	PP1	95.0	-70.0	13.5
	via5	PP1	106.1	-82.2	11.4
	I	DP1	112.9	-93.3	13.9
FPBd	O	MC3	39.9	-30.8	12.5
	via	MC1	101.3	-60.1	21.5
FPBs	Op	MC3	39.9	-30.8	12.5
	Od	MC1	43.4	-50.7	4.5
	via	MC1	101.3	-60.1	21.5
OPP	O1	MC3	34.8	-20.6	13.8
	O2	MC3	56.9	-15.3	14.1
	I*	MC1	91.2	-36.5	13.6
APB	O*	MC3	49.2	-14.0	18.8
	via	MC1	101.3	-60.1	21.5
ADPt	O*	MC3	41.5	-52.6	-3.7
	via	MC1	101.1	-67.2	4.2
ADPo	O	MC3	46.3	-27.1	4.3
	via	MC1	101.1	-67.2	4.2
APL	Op	ULN	-3.1	232.5	-0.1
	Od	RAD	10.8	155.1	2.7
	via1	RAD	60.0	13.3	8.9
	via2	MC3	61.7	-9.9	4.4
	I	MC1	79.5	-19.7	4.3
EPL	Op	ULN	-1.8	183.0	-16.3
	Od	ULN	14.8	121.5	-9.1
	via1	RAD	42.2	37.4	-8.5
	via2	MC3	45.9	2.0	-11.4
	via3	MC1	75.8	-19.2	7.9
	via4	MC1	97.2	-46.2	5.5
EPB	Op	ULN	9.8	194.9	-11.0
	Od	ULN	5.9	142.9	-13.7
	via1	RAD	49.2	28.1	0.1
	via2	MC3	64.0	-3.9	3.7
	via3	MC1	78.3	-26.8	7.1
	via4	MC1	94.7	-43.9	7.2
	I	PP1	101.0	-53.7	10.8
EM1	te1	PP1	113.5	-71.2	7.8
	te2	DP1	122.3	-87.5	9.6

"O", "via" and "I" designate origin, via and insertion points, respectively. "p", "d", "u" and "r" designate the most proximal, distal, ulnar or radial point of the origin or insertion area, respectively. * designate the middle point of the area of origin or insertion. Extrinsic extensors have no insertion point because

they insert on the extensor mechanisms on the dorsal aspect of the phalanges so that the last via point for each muscle represent the point connecting to the extensor mechanism.

Table S4d – Location of origin, insertion and via points for the index finger muscles in the neutral reference posture expressed in the ulna coordinate system.

Muscle	Point	Segment	X (mm)	Y (mm)	Z (mm)
FDP2	O1 *	ULN	-18.8	219.3	-4.2
	O2 *	RAD	9.0	154.3	8.1
	via1	RAD	36.1	-11.0	11.3
	via2	MC3	36.9	-32.3	2.3
	via3	MC2	62.5	-86.5	-10.9
	via4	PP2	67.9	-107.8	-11.4
	via5	PP2	70.1	-121.8	-11.3
	via6	MP2	75.7	-142.4	-12.1
	via7	MP2	78.9	-155.6	-13.5
	I	DP2	81.2	-172.0	-15.7
FDS2	O1p	HUM	1.6	304.7	29.3
	O1d	HUM	-4.5	291.7	32.2
	O2 *	RAD	30.3	151.3	-6.9
	via1	RAD	27.6	10.7	15.2
	via2	MC3	42.8	-31.4	6.4
	via3	MC2	58.2	-84.7	-8.3
	via4	PP2	67.5	-104.5	-9.3
	via5	PP2	70.2	-123.8	-9.7
	I1	MP2	75.3	-150.9	-13.8
	I2	MP2	81.1	-149.7	-15.4
LU1	O	MC2	41.0	-54.1	-6.8
DI1	O1p	MC1	71.0	-25.0	-3.5
	O1d	MC1	87.9	-61.2	2.4
	O2p	MC2	m.d.	m.d.	m.d.
	O2d	MC2	63.7	-62.9	-26.9
PI2	Op	MC2	43.6	-45.1	-9.4
	Od	MC2	52.1	-55.8	-13.6
EI	O	HUM	7.3	297.0	-33.4
	via1	RAD	31.3	23.7	-10.5
	via2	MC3	39.6	-25.9	-17.8
EDC	O	HUM	6.9	299.3	-31.6
	via1	RAD	31.8	21.7	-10.0
	via2	MC3	34.8	-23.8	-20.1
EM2	edc1	MC2	63.9	-80.4	-23.7
	ei	MC2	66.8	-84.2	-27.3
	edc2	PP2	70.1	-102.9	-23.3
	ui	PP2	64.5	-107.3	-14.8
	ri	PP2	73.5	-103.4	-21.0
	ub	PP2	68.3	-129.1	-16.2
	rb	PP2	81.7	-128.4	-18.7
	es1	PP2	75.2	-128.5	-21.8
	es2	MP2	76.5	-141.2	-20.7
	te1	MP2	79.9	-157.3	-20.2
	te2	DP2	80.3	-166.1	-19.9

“O”, “via” and “I” designate origin, via and insertion points, respectively. “p”, “d”, ”u” and “r” designate the most proximal, distal, ulnar or radial point of the origin or insertion area, respectively. * designate the middle point of the area of origin or insertion. Extrinsic extensors have no insertion point because they insert on the extensor mechanisms on the dorsal aspect of the phalanges so that the last via point for each muscle represent the point connecting to the extensor mechanism. “m.d.” stands for missing data.

Table S4e – Location of origin, insertion and via points for the middle finger muscles in the neutral reference posture expressed in the ulna coordinate system.

Muscle	Point	Segment	X (mm)	Y (mm)	Z (mm)
FDP	O1 *	ULN	-18.8	219.3	-4.2
	O2 *	RAD	9.0	154.3	8.1
FDP3	via1	RAD	26.5	7.7	16.7
	via2	MC3	33.6	-38.4	1.5
	via3	MC3	40.9	-85.4	-1.5
	via4	PP3	46.4	-111.9	-9.3
	via5	PP3	46.4	-133.1	-9.6
	via6	MP3	51.2	-149.1	-10.7
	via7	MP3	53.6	-164.1	-14.3
	I	DP3	53.7	-183.1	-16.5
FDS3	O1p	HUM	1.6	304.7	29.3
	O1d	HUM	-4.5	291.7	32.2
	O2 *	RAD	30.3	151.3	-6.9
	via1	RAD	21.2	9.3	14.8
	via2	MC3	34.8	-37.0	4.4
	via3	MC3	40.1	-76.6	0.3
	via4	PP3	45.6	-114.6	-10.0
	via5	PP3	46.6	-129.8	-8.5
	I1	MP3	50.6	-161.0	-13.9
	I2	MP3	56.7	-156.8	-13.0
LU2	O	MC3	36.1	-50.3	0.4
DI2	O1p	MC2	51.9	-38.5	-20.9
	O1d	MC2	57.5	-68.5	-24.2
	O2p	MC3	42.7	-35.6	-22.4
	O2d	MC3	46.1	-68.5	-20.2
DI3	O1p	MC3	33.8	-37.7	-21.1
	O1d	MC3	34.4	-64.4	-17.6
	O2p	MC4	22.1	-35.5	-15.1
	O2d	MC4	19.1	-64.6	-14.4
EM3	edc1	MC3	43.5	-83.7	-23.9
	edc2	PP3	45.0	-99.5	-22.7
	ui	PP3	37.3	-101.4	-12.9
	ri	PP3	50.8	-107.7	-18.3
	ub	PP3	43.0	-126.1	-17.1
	rb	PP3	55.2	-124.5	-18.2
	es1	PP3	49.4	-127.0	-21.3
	es2	MP3	53.8	-147.3	-21.3
	te1	MP3	55.1	-166.3	-22.0
	te2	DP3	53.9	-179.8	-22.9

“O”, “via” and “I” designate origin, via and insertion points, respectively. “p”, “d”, ”u” and “r” designate the most proximal, distal, ulnar or radial point of the origin or insertion area, respectively. * designate the middle point of the area of origin or insertion. Extrinsic extensors have no insertion point because they insert on the extensor mechanisms on the dorsal aspect of the phalanges so that the last via point for each muscle represent the point connecting to the extensor mechanism.

Table S4f – Location of origin, insertion and via points for the ring finger muscles in the neutral reference posture expressed in the ulna coordinate system.

Muscle	Point	Segment	X (mm)	Y (mm)	Z (mm)
FDP4	O1 *	ULN	-18.8	219.3	-4.2
	O2 *	RAD	9.0	154.3	8.1
	via1	RAD	24.2	3.0	12.6
	via2	MC3	30.3	-38.1	2.5
	via3	MC4	28.7	-75.7	2.6
	via4	PP4	25.3	-96.2	-4.4
	via5	PP4	16.9	-120.9	-5.5
	via6	MP4	12.2	-138.0	-5.6
	via7	MP4	13.3	-152.2	-9.1
	I	DP4	9.3	-169.9	-10.2
FDS4	O1p	HUM	1.6	304.7	29.3
	O1d	HUM	-4.5	291.7	32.2
	O2 *	RAD	30.3	151.3	-6.9
	via1	RAD	21.4	0.3	19.7
	via2	MC3	25.7	-34.2	13.9
	via3	MC4	27.7	-76.6	3.9
	via4	PP4	21.6	-99.9	-4.3
	via5	PP4	15.6	-121.3	-4.4
	I1	MP4	11.6	-144.1	-8.2
	I2	MP4	15.7	-150.4	-12.1
LU3	O	MC4	27.5	-50.3	2.1
DI4	O1p	MC4	22.3	-34.4	-14.8
	O1d	MC4	20.3	-63.9	-15.7
	O2p	MC5	11.8	-38.1	-9.9
	O2d	MC5	9.6	-60.5	-10.3
PI3	Op	MC4	28.8	-41.8	-8.2
	Od	MC4	27.3	-61.7	-8.9
EM4	edc1	MC4	25.1	-63.6	-17.5
	edc2	PP4	19.2	-88.5	-12.1
	ui	PP4	18.2	-92.7	-4.8
	ri	PP4	26.0	-96.9	-13.2
	ub	PP4	11.6	-114.2	-9.1
	rb	PP4	21.6	-119.2	-15.1
	es1	PP4	15.3	-119.8	-15.9
	es2	MP4	13.1	-133.9	-14.9
	te1	MP4	14.4	-156.1	-15.2
	te2	DP4	10.2	-164.8	-15.9

“O”, “via” and “I” designate origin, via and insertion points, respectively. “p”, “d”, ”u” and “r” designate the most proximal, distal, ulnar or radial point of the origin or insertion area, respectively. * designate the middle point of the area of origin or insertion. Extrinsic extensors have no insertion point because they insert on the extensor mechanisms on the dorsal aspect of the phalanges so that the last via point for each muscle represent the point connecting to the extensor mechanism.

Table S4g – Location of origin, insertion and via points for the little finger muscles in the neutral reference posture expressed in the ulna coordinate system.

Muscle	Point	Segment	X (mm)	Y (mm)	Z (mm)
FDP	O1 *	ULN	-18.8	219.3	-4.2
	O2 *	RAD	9.0	154.3	8.1
FDP5	via1	RAD	18.3	3.2	9.9
	via2	MC3	23.5	-37.8	2.2
	via3	MC5	14.6	-72.1	6.0
	via4	PP5	10.9	-88.9	3.2
	via5	PP5	5.2	-108.8	0.8
	via6	MP5	1.2	-126.4	2.7
	I	DP5	2.0	-144.4	1.8
FDS5	O1p	HUM	1.6	304.7	29.3
	O1d	HUM	-4.5	291.7	32.2
	O2 *	RAD	30.3	151.3	-6.9
	via1	RAD	11.8	1.8	13.6
	via2	MC3	23.3	-25.6	16.5
	via3	MC5	17.0	-69.8	8.1
	via4	PP5	6.8	-98.4	-0.1
	via5	PP5	4.6	-106.6	-0.8
	I1	MP5	-0.1	-125.2	2.5
	I2	PP5	6.2	-125.3	2.8
LU4	O	MC5	23.0	-51.4	6.6
PI4	Op	MC5	17.4	-43.1	-3.4
	Od	MC5	12.3	-61.5	-2.8
FDM	O	MC5	29.9	-30.0	13.1
	I	PP5	-4.8	-96.5	5.0
ADM	O	MC5	24.3	-22.1	13.7
	I	PP5	-4.8	-96.5	5.0
ODM	O	MC3	25.9	-27.1	12.1
	Ip	MC5	10.2	-43.5	-0.6
	Id	MC5	7.2	-62.5	-2.5
EDM	O	ULN	0.9	158.1	-15.0
	via1	RAD	14.4	23.0	-0.7
	via2	MC3	14.0	-7.1	-0.4
EDC	O	HUM	6.9	299.3	-31.6
	via1	RAD	31.8	21.7	-10.0
	via2	MC3	34.8	-23.8	-20.1
EM5	edm	MC5	9.3	-55.5	-10.2
	edc1	MC5	21.8	-60.3	-16.5
	edc2	PP5	2.4	-81.8	-4.0
	ui	PP5	-1.4	-87.4	8.9
	ri	PP5	9.7	-90.8	-3.9
	ub	PP5	-2.0	-105.8	2.3
	rb	PP5	7.4	-111.0	-5.7
	es1	PP5	1.5	-108.1	-5.7
	es2	MP5	0.8	-116.0	-4.7
	te1	MP5	-1.0	-134.2	-1.2
	te2	DP5	1.5	-142.9	0.2

“O”, “via” and “I” designate origin, via and insertion points, respectively. “p”, “d”, ”u” and “r” designate the most proximal, distal, ulnar or radial point of the origin or insertion area, respectively. * designate the middle point of the area of origin or insertion. Extrinsic extensors have no insertion point because they insert on the extensor mechanisms on the dorsal aspect of the phalanges so that the last via point for each muscle represent the point connecting to the extensor mechanism.

Table S5a – Muscle morphological parameters for forearm and hand extrinsic muscles measured after fixation of muscle.

	Mass (g)	PCSA (cm^2)	Penn. angle (°)	Belly length (mm)	Fibre length (mm)	Index of architecture (d.u.)	Tendon length (mm)
PRT	51.4	6.8	35	193	72	0.37	/
PRQ	13.7	3.9	53	66	34	0.51	/
SUP	26.9	7.5	0	73	34	0.47	/
PL	12.0	1.5	22	206	76	0.37	239
FCU	62.1	7.3	35	394	81	0.20	239
FCR	32.3	3.9	18	251	78	0.31	209
ECU	42.4	8.9	35	255	45	0.18	249
ECRL	55.1	6.1	42	163	86	0.53	234
ECRB	41.9	6.2	33	182	64	0.35	217
FPL	21.8	2.5	30	260	81	0.31	260
APL	18.6	3.0	29	212	58	0.27	169
EPL	8.2	1.4	0	173	55	0.32	152
EPB	4.6	0.7	30	215	59	0.27	56
FDSI	24.8	3.4	40	287	69	0.24	266
FDSM	41.2	4.9	31	274	79	0.29	297
FDSR	16.5	1.6	25	230	98	0.43	261
FDSL	5.9	0.6	0	313	102	0.33	186
FDPI	39.2	4.0	0	280	92	0.33	300
FDPM	43.7	3.7	0	277	113	0.41	340
FDPR	24.5	2.1	0	274	111	0.41	305
FDPL	18.7	1.9	0	249	95	0.38	288
EDCI	8.7	1.3	0	206	62	0.30	183
EDCM	17.4	2.7	0	206	62	0.30	176
EDCR	13.1	2.0	20	243	61	0.25	242
EDCL	6.5	1.1	20	144	56	0.39	244
EI	6.8	1.2	14	122	52	0.42	246
EDM	6.5	1.1	22	132	58	0.43	162

Table S5b – Muscle morphological parameters for hand intrinsic muscles measured after fixation of muscle.

	Mass (g)	PCSA (cm²)	Penn. angle (°)	Belly length (mm)	Fibre length (mm)	Index of architecture (d.u.)	Tendon length (mm)
FPBs	7.3	1.3	0	81	52	0.64	/
FPBd	3.8	1.1	0	85	33	0.39	/
APB	6.2	1.2	0	74	47	0.64	29
OPP	2.8	0.7	0	86	40	0.46	/
ADPt	6.2	1.6	0	71	37	0.52	/
ADPo	10.8	3.6	0	81	28	0.35	/
LU1	1.1	0.1	0	110	77	0.70	/
LU2	1.3	0.2	0	116	74	0.64	/
LU3	1.1	0.2	0	112	68	0.61	/
LU4	0.9	0.1	0	104	76	0.74	/
PI2	2.4	0.8	0	59	29	0.50	/
PI3	3.0	1.1	0	64	25	0.39	/
PI4	2.5	0.8	0	54	29	0.54	/
DI1	12.6	3.0	0	92	39	0.43	/
DI2	2.2	0.8	0	41	25	0.62	/
DI3	3.5	1.5	0	74	21	0.29	/
DI4	3.1	1.5	0	46	20	0.43	/
FDM	2.2	0.7	0	73	29	0.40	/
ODM	3.4	1.4	0	107	23	0.21	/
ADM	8.4	1.4	0	96	56	0.58	/

Table S6 – Parameters of cylinders used to constraint the tendon paths. The point coordinates and the radii are provided in mm.

		Point			Axis			
		X	Y	Z	X	Y	Z	Radius
FCU	Cylinder 1 (flex)	25.9	-256.9	50.2	-0.971	-0.227	-0.081	4.0
	Cylinder 2 (ext)	30.3	-273.2	33.3	-0.971	-0.227	-0.081	12.0
FCR	Cylinder 1 (flex)	28.8	-269.1	50.8	-0.971	-0.227	-0.081	2.5
	Cylinder 2 (ext)	31.3	-277.9	34.9	-0.971	-0.227	-0.081	12.0
PL	Cylinder 1 (flex)	25.3	-254.7	55.9	-0.971	-0.227	-0.081	5.0
	Cylinder 2 (ext)	29.9	-272.0	35.6	-0.971	-0.227	-0.081	15.0
ECU	Cylinder 1 (flex)	30.3	-273.8	36.9	-0.971	-0.227	-0.081	10.0
	Cylinder 2 (ext)	29.5	-269.3	25.7	-0.971	-0.227	-0.081	1.5
ECRL	Cylinder 1 (flex)	30.9	-275.5	30.6	-0.971	-0.227	-0.081	10.0
	Cylinder 2 (ext)	29.6	-269.2	20.6	-0.971	-0.227	-0.081	1.5
ECRB	Cylinder 1 (flex)	30.8	-275.0	27.0	-0.971	-0.227	-0.081	10.0
	Cylinder 2 (ext)	29.0	-266.2	18.7	-0.971	-0.227	-0.081	1.5
FCU	Cylinder 1 (flex)	29.1	-269.0	39.7	-0.971	-0.227	-0.081	1.0
	Cylinder 2 (ext)	30.7	-275.1	34.0	-0.971	-0.227	-0.081	5.0
